# Chicken Manure and Mushroom Residues Affect Soil Bacterial Community Structure but Not the Bacterial Resistome When Applied at the Same Rate of Nitrogen for 3 Years

**DOI:** 10.3389/fmicb.2021.618693

**Published:** 2021-05-21

**Authors:** Shuang Peng, Yiming Wang, Ruirui Chen, Xiangui Lin

**Affiliations:** ^1^College of Environment and Ecology, Jiangsu Open University, Nanjing, China; ^2^State Key Laboratory of Soil and Sustainable Agriculture, Institute of Soil Science, Chinese Academy of Sciences, Nanjing, China; ^3^Jiangsu Collaborative Innovation Center for Solid Organic Waste Resource Utilization, Nanjing, China

**Keywords:** high-throughput quantitative polymerase chain reaction, antibiotic resistance, chicken manure, bacterial communities, mushroom residue

## Abstract

Animal manure is a reservoir of antibiotic resistance genes (ARGs), and direct application of the manure will lead to spread of ARGs in farmland. Here, we explored the impacts of chicken manure and heat-treated chicken manure on the patterns of soil resistome after 3 years’ application, with mushroom residues set as the plant-derived organic manure treatment. A total of 262 ARG subtypes were detected in chicken manure using high-throughput qPCR, and heat treatment can effectively remove 50 types of ARGs. Although ARG subtypes and abundance were both higher in chicken manure, there was no significant difference in the ARG profiles and total ARG abundance among three manure-treated soils. Soil bacteria community compositions were significantly different among manure-treated soils, but they were not significantly correlated with soil ARG profiles. Fast expectation–maximization microbial source tracking (FEAST) was used for quantifying the contributions of the potential sources to microbial taxa and ARGs in manure-fertilized soil. Results revealed that only 0.2% of the chicken manure-derived bacterial communities survived in soil, and intrinsic ARGs were the largest contributor of soil ARGs (95.8–99.7%); ARGs from chicken manure only contributed 0.4%. The total ARG abundance in the heat-treated chicken manure-amended soils was similar to that in the mushroom residue-treated soils, while it was 1.41 times higher in chicken manure-treated soils. Thus, heat treatment of chicken manure may efficiently reduce ARGs introduced into soil and decrease the risk of dissemination of ARGs.

## Introduction

The increasing emergence and spread of antibiotic resistant bacteria (ARB) and antibiotic resistance genes (ARGs) are of great concern worldwide, threatening the efficacy of modern medicines and posing risks to human health (Levy and Marshall, 2004; Su et al., 2015). ARB and ARGs have been shown to be ubiquitous in the natural environment, agricultural soil (Wang et al., 2020a), freshwater (Xiong et al., 2015), sediments (Guo et al., 2020), human gut (McInnes et al., 2020), and organic manure (Peng et al., 2018) contain distinct ARG contents at varying abundances. Environmental microbiome is now recognized as a reservoir of ARGs observed in the clinical setting; some plasmid-mediated ARGs spread globally in recent decades have been traced to environmental and animal origins (Lee et al., 2020). Environmental research to better understand distribution and transmission of ARGs may help to reduce the anthropogenic influences on the environmental resistome.

Soil harbors a large genetic diversity at small spatial scale, favoring exchange of genetic materials by means of horizontal gene transfer that will contribute to ARG dissemination (Nesme and Simonet, 2015). The transfer of ARGs from soil bacteria to human pathogens could bring about tremendous threats to human disease control and prevention (Forsberg et al., 2012). ARGs could be transferred from soil to the plant microbiomes (Zhang et al., 2019), which is one of the ways to enter the human body. Livestock manure is the main source of ARB and ARGs in the agriculture soil environment (Peng et al., 2017; Qian et al., 2017). Previous studies have demonstrated that fertilization with pig manure or chicken manure led to an increase in soil ARGs (Peng et al., 2017; Urra et al., 2019). The composting process minimizes some chemical and biological risks (e.g., presence of pathogens) (Evanylo et al., 2008), so this option is commonly preferred before livestock manure is applied. However, compost could still lead to the increased amount of ARGs in soil (Zhu et al., 2013; Peng et al., 2015; Sun et al., 2019). ARB and ARGs in composted manure can spread and promote further development of antibiotic resistance (Kang et al., 2016; Wang et al., 2020a). Therefore, there is a vital need to further improve the effect of compost practice on ARGs carried by animal feces, monitor ARGs in manure-treated soils, and further examine manure application practices and impacts.

Organic manure originated from plants rather than animals, usually used to ameliorate soil properties, both abiotically and biotically (Feng et al., 2015). Mushroom substrate residues is rich in cellulose, lignin, vitamins, and other bioactive substances; rational utilization of mushroom residues is conducive to environmental protection and sustainable development of agriculture (Li et al., 2020). Long-term fertilization with chemical fertilizers plus mushroom residues showed a more connective and closer bacterial networks compared with chemical fertilization (Yao et al., 2020). Recent studies about ARGs mainly shed light on the dynamics of ARGs during manure treatment process (Gou et al., 2018; Awasthi et al., 2019; Wu et al., 2020) and in agricultural soils following application of animal manures (Deng et al., 2020; Liu et al., 2021). Little attention was paid to ARGs in plant-derived organic manure, which also contain a huge number of microorganisms. A previous study found that long-term (22 years) fertilization with plant-derived organic manure increased the abundance of *tetL* and *intI1* in soil (Peng et al., 2018). Mushroom residues are a kind of organic manure completely different from animal manure; their effect on soil microbial community was also quite different (Yao et al., 2020). Analyzing the effect of long-term application of mushroom residues on soil antibiotic resistome may broaden our horizons about how organic manures affect soil antibiotic resistome.

Many factors can influence the fate and migration of manure-borne ARGs in the environment. Studies showed that variations in ARGs composition were mainly structured by phylogenetic compositions of microbial communities (Forsberg et al., 2014; Su et al., 2015; Chen et al., 2016; Liu et al., 2017). Mobile genetic elements (MGEs), such as gene cassettes, integrons (capture ARGs), plasmids (conjugation and transfer ARGs), transposons, and insertion sequences (transfer ARGs) may also influence the spread of ARGs in the environment (Zhang et al., 2011). Chicken manure and mushroom residues are two completely different fertilizers: one originates from animal feces, while the other derives from plant, so they may composite completely different microbial communities and MGEs. In addition, whether sterilization of chicken manure with high-temperature can reduce the risk of ARGs contamination is still unknown. Understanding how these manures contribute to development of soil antibiotic resistome under long-term application is essential since it may direct measures ultimately aiming to diminish risks. The aim of this work was to study and contrast the impact of the 3 years’ application of different manures, according to the origin (chicken manure-derived vs. mushroom residues) and sterile conditions (chicken manure vs. heat-treated chicken manure), on ARG and MGE composition in agricultural soil, as well as their relationship with bacteria communities. Studies have reported that partial replacement of chemical fertilizers with organic fertilizers could increase vegetable or grain yields and improve quality of soil and product (Wang et al., 2020b; Yi et al., 2021). In this study, different manures were used to replace 50% of chemical fertilizer, in the case of the same amount of nitrogen applied to the soil. Results from the study will be helpful in estimating the environmental loads of ARGs derived from different manures to soil, understanding how these fertilizers influence soil resistome and guiding manure application practices with the same amount of N input.

## Materials and Methods

### Description of Experiment Site and Different Organic Manure

The long-term experiment field is located at the Fengqiu Agroecological Experimental Station (35°00′N, 114°24′E) in Henan Province, China. The soil is classified as Aquic Inceptisol according to United States Department of Agriculture (USDA), derived from alluvial sediments of the Yellow River, with a sandy loam texture. The long-term experiment was initiated in 2011 with different fertilization treatments rotated with winter wheat (*Triticum aestivum* L.) and summer maize (*Zea mays* L.). The treatments were arranged in a randomized block design, and each treatment had four replicated plots. These experimental plots, designed originally for a nutrient cycling study, enabled us to also examine the effect of manure application on the profiles of soil ARGs.

In the present study, we used four fertilizer treatments and one unfertilized control (CK). The fertilizer treatments included nitrogen (N), phosphorus (P), and potassium fertilizers (F) and the combined application of NPK fertilizers with mushroom residues (MRF) or with chicken manure (CMF) or heat-treated chicken manure (KF). For the F treatment, N, P, and potassium were applied in the form of urea (200 kg/ha N), superphosphate (80 kg/ha P_2_O_5_), and potassium sulfate (150 kg/ha K_2_O), respectively. The MRF, CMF, and KF treatments were designed to supply the same rate of total N with F: one-half of N came from urea and the other half was from mushroom residues, chicken manure, or heat-treated chicken manure. Chicken manure (CM) and mushroom residues (MR) were collected from nearby farms. Chicken manure was treated at 260°C for 4 h in an oil bath high-temperature treatment kettle to turn it into heat-treated manure (K). The organic and inorganic fertilizers were applied before sowing in June for maize and in October for wheat. The area of each plot was 30 m^2^, and more information was described by Yao et al. (2020).

### Soil Sampling and DNA Extraction

Soil samples were collected on September 2014, after the maize harvest. At each sampling plot, soils were collected from 0- to 20-cm layer at six random locations; the plant residues and visible roots were removed, mixed to homogenize inside a plastic bag representing the sample of the plot, and carried to lab kept on ice. The mushroom residues and chicken manure to be applied in the field during 2014 were also sampled, and three replicates were collected for analysis. Approximately 10-g soil samples or manure were stored at −80°C for DNA extraction; the rest of the soil samples were air dried and used for chemical analysis as described by Yao et al. (2020).

The microbial genomic DNA was extracted from 0.50-g soil or manure sample using the FastDNA^®^ SPIN Kit for soil or for feces (MP Biomedicals, Santa Ana, CA, United States) according to the manufacturer’s protocol. DNA concentration was determined on a ND-1000 spectrophotometer (Thermo-Scientific, Wilmington, DE, United States), and the extracted DNA was stored at −20°C until use. The DNA samples were diluted to 10× and 50× to avoid inhibitors to the PCR.

### qPCR Analysis of Antibiotic Resistance Genes and Mobile Genetic Elements

High-throughput quantitative PCRs (HT-qPCR) were conducted using WaferGen SmartChip Realtime PCR system that contained a total of 370 validated primer sets as reported previously (Muurinen et al., 2017). A threshold cycle value (*C*_*T*_) of 31 was used as the detection limit to differentiate between positive amplification and primer–dimers (Su et al., 2015). One negative control with no DNA template added was included in each HT-qPCR run to eliminate false-positive detections. Amplicons with multiple melting curves were removed from the analysis. Three technical replicates of each sample above the detection limit were regarded as positive detection. The 2^–Δ^*^CT^* method where Δ*C*_T_ = (*C*_*T*_ detected ARGs - *C*_*T*_ 16S rRNA gene) was used to calculate the relative abundances of ARGs and MGEs normalized to the 16S rRNA gene according to a comparative *C*_*T*_ method (Gou et al., 2018).

Fifteen genes of special concern ARGs including tetracycline resistance genes [*tetG*, *tetL*, tet*Z*, *tetW*, *tetM*, *tetO*, and *tetB*(*P*)], sulfonamide resistance genes (*sul1*, *sul2*, and *sul3*), macrolide resistance genes (*ermB*, *ermC*, and *ermF*), and beta-lactamase genes (*bla*_*CTX*_ and *bla*_*TEM*_) were quantified by real-time quantitative PCR (RT-qPCR) analysis with a C1000^TM^ Thermal Cycler equipped with the CFX96^TM^ Real-Time system (Bio-Rad, United States). In addition, the 16S rRNA gene abundance, which has been used previously to assess the overall bacterial abundance, was quantified by using primer set 519F/907R so that the ARG abundance could be standardized against the bacterial populations. The qPCR system and primers sets as reported previously (Peng et al., 2017).

### High-Throughput Sequencing of 16S rRNA Gene

The 16S rRNA gene sequencing was performed at Novegene (Beijing, China) using the Ion S5^TM^ XL platform, and 400-bp/600-bp single-end reads were generated. The 16S V4 region was amplified using the primers 515F (5′-GTGCCAGCMGCCGCGGTAA-3′) and 806R (5′-GGACTACVSGGGTATCTAAT-3′).

Single-end reads were assigned to samples based on their unique barcode and truncated by cutting off the barcode and primer sequence. Quality filtering on the raw reads was performed under specific filtering conditions to obtain high-quality clean reads according to the Cutadapt (Martin, 2011) (V1.9.1) quality-controlled process. The reads were compared with the reference database (Gold database) using the UCHIME algorithm (Edgar et al., 2011) to detect chimera sequences, and then the chimera sequences were removed. Then the effective tags were finally obtained. Sequences analysis was performed by Uparse software (Uparse v7.0.1001). Operational taxonomic units (OTUs) were defined at the 97% similarity level. A representative sequence of each OTU was assigned to a taxonomic level in the SILVA database. The sequences were deposited into the National Center for Biotechnology Information (NCBI) Sequence Read Archive (SRA) database (accession no., PRJNA669308).

### Manure and Mushroom Residue-Associated Community and Antibiotic Resistance Gene Analyses

Fast expectation–maximization microbial source tracking (FEAST) was used to calculate the contribution of soil, mushroom residues, and chicken manure bacterial communities within treatments following the procedures as described previously (Shenhav et al., 2019) with the R package of ‘‘FEAST^1^.” FEAST could unravel the origins of complex microbial communities based on the statistical model that assumes each sink is a convex combination of known and unknown sources. In this study, the chicken manure, mushroom residues, and unfertilized soil samples were defined as manure or soil “sources” and manure-treated soil samples as “sinks.” OTUs present in only one sample were removed prior to the analysis, and the algorithm was run with default parameters. In order to understand the source–sink relationship in fertilized soil, FEAST was also used to predict the relative contribution of different manures to the ARGs in treated soil based on the signatures of ARGs as described previously (Chen et al., 2020).

### Statistical Analysis

The effects of different manure fertilization on abundance of ARGs and soil chemical properties were compared using ANOVA performed with software SPSS 20 (IBM, Armonk, NY, United States). Tukey’s honestly significant difference (HSD) test was used for comparisons among treatment means. Differences were considered significant at *p* < 0.05. The changes of ARGs and bacterial compositions across different treatments were visualized by principal coordinate analysis (PCoA) and non-metric multidimensional scaling (NMDS) ordinations based on the Bray–Curtis dissimilarity distances. Significant difference tests in the bacterial communities and profiles of ARGs and MGEs between different treatments were conducted using ANOSIM and nonparametric permutational multivariate analysis of variance (PERMANONA, with “adonis” function) based on Bray–Curtis dissimilarities and 999 permutations. The Procrustes analysis (9,999 permutations) was used to describe the correlation between ARGs composition and bacterial communities. The above statistical analysis and redundancy analysis (RDA) were processed and plotted by R version 3.6.3 using the package vegan. Heatmap was performed with the R package pheatmap. Additionally, we tested for differential ARG abundance between manure-treated soil and chemical fertilizer treated soil (F) using likelihood ratio tests (LRTs) with the R package edgeR at a false discovery rate (FDR)-corrected value of *p* < 0.05 and exhibited with Manhattan plots based on R packages of ggplot2. Network maps based on the Spearman correlation coefficient between ARGs and MGEs were drawn using the Gephi platform based on a significant Spearman correlation (Spearman’s rho > 0.9, *P*_adjusted_ < 0.01).

## Results

### Diversity and Relative Abundance of Antibiotic Resistance Genes and Mobile Genetic Elements in Manure

**FIGURE 1:**
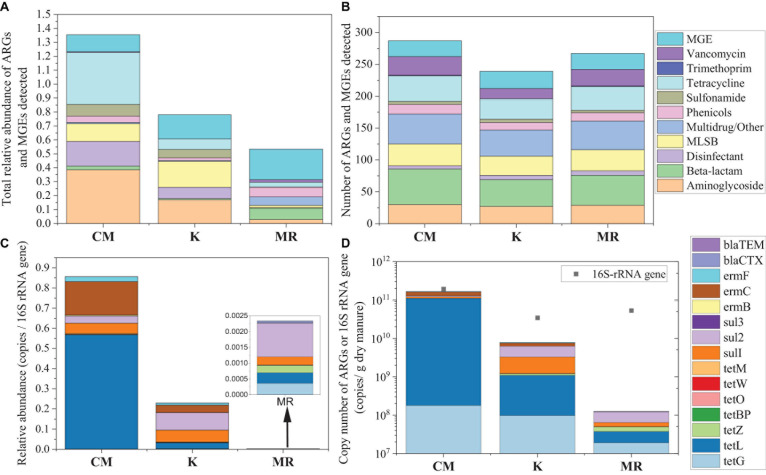
Comparison of the average total relative abundance **(A)** and the major classes **(B)** of ARGs and MGEs in manure samples quantified by high-throughput quantitative PCR (HT-qPCR). The average relative abundances **(C)** and copy numbers **(D)** of 13 ARGs or 16S-rRNA gene in manure samples quantified by real-time quantitative PCR (RT-qPCR). CM, chicken manure; K, heat-treated chicken manure; and MR, mushroom substrate residue; ARGs, antibiotic resistance genes; MGEs, mobile genetic elements.

The types and abundance of ARGs were analyzed by HT-qPCR in manure samples. We detected 10, 10, and 9 ARG types in manure of CM, MR, and K, respectively (Figure 1A). A total of 262 ARG subtypes were detected in CM, which was more than those in MR (242) and K (212) (Figure 1A). The total relative abundance of ARGs in CM was approximately 2.03 times and 3.92 times higher than that in K and MR (Figure 1B).

Compositions of ARGs in these three manures were also quite different. The main ARGs in CM belong to aminoglycoside, tetracycline, disinfectant, and sulfonamide, while MR was dominated by ARGs that belong to beta-lactam, multidrug/other, and phenicols (Figure 1B). MLSB resistance genes were the main residual ARGs in manure K. A different pattern was found for MGEs, with an average number of 25 MGEs detected in CM and K, while MR harbored averagely 27 MGEs. The total relative abundance of MGEs was higher in MR than that in CM and K, but the difference was not significant (*p* > 0.05) (Figure 1B).

Fifteen ARGs were quantified with RT-qPCR, and the result was consistent with that of HT-qPCR, while the difference among these three manures is more obvious (Figures 1C,D). The total relative abundance of the 15 ARGs in CM was 3.73 and 364.95 times higher than that in K and MR (Figure 1C). The total copy number of ARGs in MR was also lower than that of CM and K, with 16S rRNA gene copy number at the same level as K (Figure 1D).

### Profiles of Antibiotic Resistance Genes in Soils After Different Manures Applied for 3 Years

**FIGURE 2:**
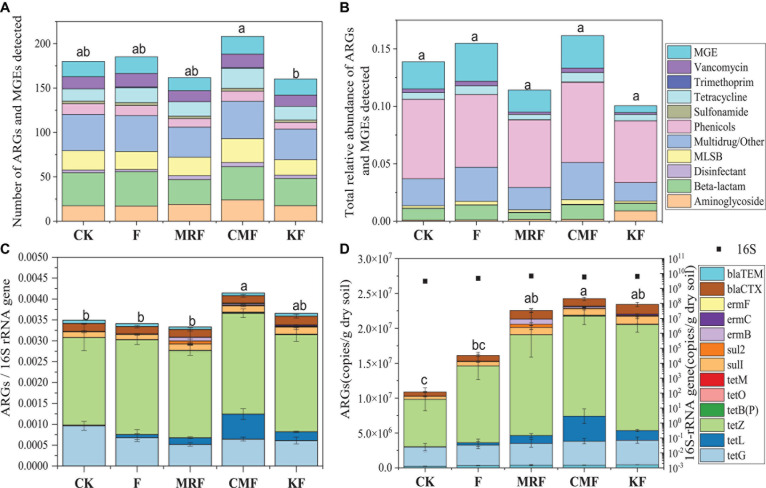
Number of unique ARGs and MGEs detected in the soil sample of each treatment **(A)** and the average relative abundance of ARGs and MGEs in soil fertilized with each manure for 3 years **(B)** quantified by HT-qPCR. The average relative abundance **(C)** or copy numbers **(D)** of 13 ARGs in soil samples quantified by RT-qPCR. Different letters indicate significant differences based on Tukey’s HSD test at *p* < 0.05. Error bars represent standard deviation (*n* = 4). CK, unfertilized control; F, NPK fertilizers; KF, the combined application of NPK fertilizers and heat-treated chicken manure; CMF, the combined application of NPK fertilizers and chicken manure; MRF, the combined application of NPK fertilizers and mushroom residues; ARGs, antibiotic resistance genes; MGEs, mobile genetic elements; HSD, honestly significant difference.

A total of 193 ARG subtypes were detected in CMF soils, 152 detected in MRF soils, and 146 detected in KF soils. The number of ARG subtypes detected in MRF and KF was less than that in F soils (172) (Figure 2A). Most of these ARGs represented three resistance mechanisms: antibiotic deactivation (38%), efflux pump (36%), and cellular protection (20%) (Supplementary Figure 1). Phenicols and multidrug/other resistance genes were the dominant ARG types in soils (Figure 2B). Among three manure treatments, CMF soils had higher total relative abundance of ARGs and MGEs, but neither of the differences reached the significant level (*p* > 0.05) (Figure 2B).

**FIGURE 3:**
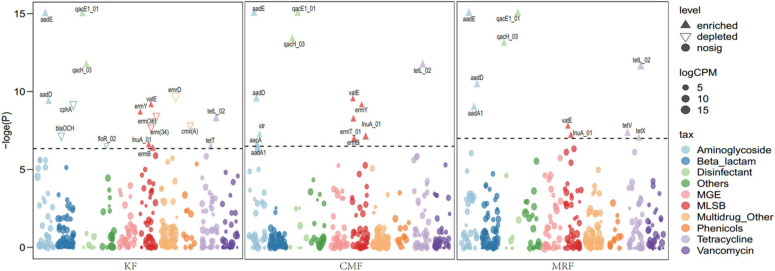
Manhattan plots showing manure-enriched ARGs in KF, CMF, or MRF compared with F. ARGs that are significantly enriched (with respect to F) are depicted as full triangles; solid dots marked with “nosig” represent no significant difference. The color of each dot represents ARG types or MGE, and the size corresponds to their relative abundances in the respective samples. ARGs belonging to sulfonamide and trimethoprim are labeled as “Others.” F, NPK fertilizers; KF, the combined application of NPK fertilizers and heat-treated chicken manure; CMF, the combined application of NPK fertilizers and chicken manure; MRF, the combined application of NPK fertilizers and mushroom residues; ARGs, antibiotic resistance genes; MGE, mobile genetic element.

Many ARGs were enriched in the soils fertilized with KF, CMF, or MRF (Figure 3 and Supplementary Figure 2). Among them, seven ARGs, including *aadE*, *aadD*, *qacE1*, *qacH*, *lnuA*, *vatE*, and *tetL*, were significantly enriched in all three manure treatments. Aminoglycoside resistance genes *aadA1* were enriched in CMF and MRF, while MLSB resistance genes *ermB* and *ermY* were enriched in CMF and KF (Figure 3). There were some specific ARGs enriched in only one treatment, such as *tetV* and *tetX* in MRF soils; *aacA*, *str*, and *ermT* in CMF soils; and *tetT* in KF soils. Seven ARGs depleted in KF, including *blaOCH*, *cphA*, *erm*(*34*), *erm*(*36*), *emrD*, *cmx*(*A*), and *floR* (Figure 3). PCoA and ANOSIM pairwise comparisons of the overall distribution patterns of ARGs and MGEs in soils demonstrated no distinct separation among these treatments (Supplementary Figure 3 and Supplementary Table 1). Network maps based on the Spearman correlation coefficient between ARGs and MGEs showed that manure fertilization reduced the network connection between different ARGs and between ARG and MGEs (Supplementary Figure 4). The connection edge number in KF treatment was the least among the four fertilization treatments (Supplementary Figure 4).

Fifteen ARGs were quantified by RT-qPCR, while *tetW* and *sul3* were not detectable in all of the soil samples. The total relative abundance of the detected 13 ARGs in CMF soils was significantly higher than that of MRF and F soils (*p* < 0.05) (Figure 2C), while that in KF and MRF was similar with each other (*p* > 0.05) (Figure 2D). In Figures 2C,D, CMF soils had obviously a higher abundance of *tetL*. Statistical analysis showed that *tetL*, *tetB*(*P*), *tetO*, and *tetM* were significantly enriched in CMF soils (Supplementary Figure 5). However, in results presented by HT-qPCR, *tetB*(*P*), *tetO*, and *tetM* were not significantly enriched in CMF soils (Figure 3).

### Soil Chemical Properties and Changes in Soil Bacterial Communities

Different fertilization treatments significantly affected the soil chemical properties (Table 1). Compared with F, MRF treatment significantly increased the concentrations of soil organic carbon, total N, total P, available P, and available potassium (*p* < 0.05), while CMF treatment significantly increased the soil contents of total N and available P (*p* < 0.05). Except that the available P was higher in CMF-treated soil (*p* < 0.05), there was no significant difference in the soil chemical properties between KF and CMF treatments. Based on the above differences of soil chemical nutrients, MRF-treated plots obtained the highest maize yield (Table 1), while there was no significant difference among CMF, KF, and F treatments.

**TABLE 1:** The yield of maize and soil chemical properties in different treatments.

	**CK**	**F**	**MRF**	**CMF**	**KF**
Yield of maize (kg/plot)	12.45 ± 0.43c	31.83 ± 0.74ab	33.68 ± 0.92a	31.42 ± 1.26b	31.04 ± 1.12b
pH	8.51 ± 0.06a	8.44 ± 0.05a	8.44 ± 0.08a	8.49 ± 0.02a	8.47 ± 0.03a
Soil organic carbon (SOC) (g/kg)	6.12 ± 0.16c	6.71 ± 0.26bc	10.84 ± 1.02a	7.7 ± 0.22b	7.44 ± 0.16b
Total N (g/kg)	0.6 ± 0.01d	0.67 ± 0.02cd	0.99 ± 0.07a	0.78 ± 0.03b	0.75 ± 0.03bc
Total P (g/kg)	0.77 ± 0.03c	0.87 ± 0.02bc	0.99 ± 0.09a	0.97 ± 0.03ab	0.9 ± 0.06ab
Available P (mg/kg)	0.52 ± 0.18d	4.28 ± 0.99d	48.07 ± 8.04a	28.86 ± 5.7b	17.93 ± 4.91c
Total potassium (g/kg)	19.23 ± 0.27a	19.64 ± 0.12a	19.62 ± 0.41a	19.46 ± 0.45a	19.6 ± 0.18a
Available potassium (mg/kg)	77.12 ± 3.94c	127.73 ± 18.25b	191.6 ± 12.68a	138.58 ± 10.69b	122.91 ± 4.82b

*Different lowercase letters indicate statistically significant differences at *p* < 0.05. CK, unfertilized control; F, NPK fertilizers; MRF, the combined application of NPK fertilizers and mushroom residues; CMF, the combined application of NPK fertilizers and chicken manure; KF, the combined application of NPK fertilizers and heat-treated chicken manure.*

**FIGURE 4:**
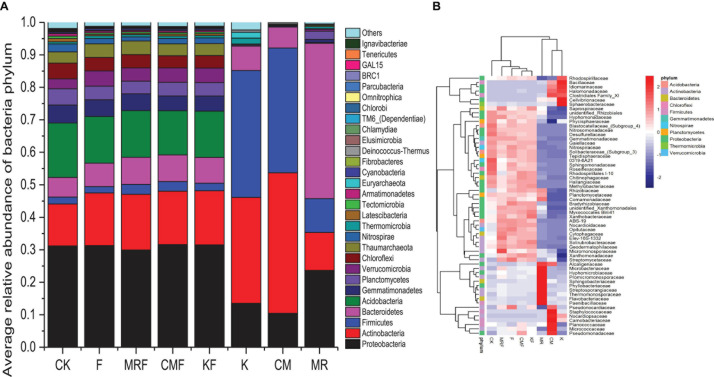
The relative abundance of 16S rRNA gene sequences classified to each phylum **(A)** and heatmap of the top 60 bacterial families **(B)** in soil samples from different treatments and manures. CK, unfertilized control; F, NPK fertilizers; KF, the combined application of NPK fertilizers and heat-treated chicken manure; CMF, the combined application of NPK fertilizers and chicken manure; MRF, the combined application of NPK fertilizers and mushroom residues; K, heat-treated chicken manure; CM, chicken manure; MR, mushroom residues. Each color block represents the mean of four replicates.

Manure bacterial communities were dominated by Firmicutes, Proteobacteria, Actinobacteria, and Bacteroidetes (Figure 4A). The total relative abundances of these bacterial phyla in manure of K, CM, and MR, respectively, accounted for 92.7, 98.6, and 93.5% of the total bacterial 16S rRNA gene sequences (Figure 4A). Phylum-level relative abundance data showed that bacterial community composition in MR was obviously different with that in CM and K. MR were dominated by the phyla Bacteroidetes (47.8%), Proteobacteria (23.7%), Actinobacteria (11.7%), and Firmicutes (10.4%), while the bacterial communities in CM and K samples were dominated by Actinobacteria (43.3 vs. 32.6%), Firmicutes (38.4 vs. 39.1%), Proteobacteria (10.4 vs. 13.5%), and Bacteroidetes (6.4 vs. 7.5%). However, the soil bacteria community structures treated by different manures were similar with each other, with Proteobacteria (29.9–31.6%) tending to be the most abundant bacterial phylum followed by Acidobacteria (13.6–16.7%) and Actinobacteria (12.9–17.1%) (Figure 4A). Although the overall bacterial community structures in the phylum level seem similar with each other in soils (Figure 4A), the application of different manures still led to changes in the abundance of some bacterial families (Figure 4B). Many special bacterial families were enriched in manure-amended soils, such as Pseudomonadaceae, Micrococcaceae, and Planococcaceae in CMF soils (Figure 4B).

**FIGURE 5:**
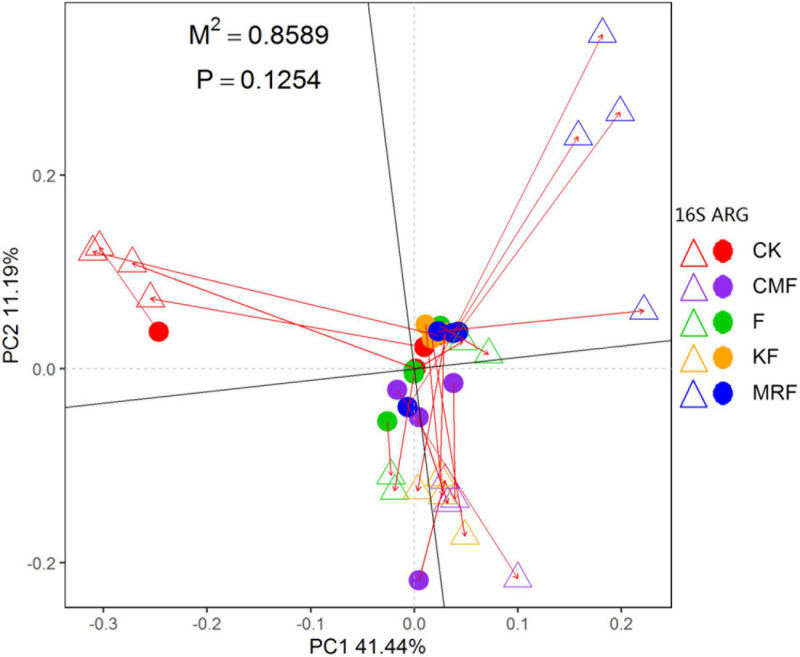
Procrustes analysis results depicting the correlation between ARG contents (HT-qPCR data) and bacterial compositions (16S rRNA gene OTU data) on the basis of Bray-Curtis dissimilarity metrics (sum of squares *M*^2^ = 0.8589, *r* = 0.3756, *p* > 0.05, 9,999 permutations). The solid dot represents ARG profiles, and the triangles indicate bacterial community. CK, unfertilized control; F, NPK fertilizers; KF, the combined application of NPK fertilizers and heat-treated chicken manure; CMF, the combined application of NPK fertilizers and chicken manure; MRF, the combined application of NPK fertilizers and mushroom residues; ARG, antibiotic resistance gene; OTU, operational taxonomic unit.

The NMDS ordination based on Bray–Curtis distances showed that bacterial communities were separated between manure treatments and CK (Supplementary Figure 6). ANOSIM pairwise comparisons were all significantly different except CMF vs. KF (Supplementary Table 2). RDA was conducted to determine the correlation of soil properties with bacterial community structures in the soils. All soil properties together explained 50.3% of the variety in soil bacterial communities. Soil organic carbon (*p* = 0.002), total N (*p* = 0.009), pH (*p* = 0.011), and total P (*p* = 0.037) were significantly correlated with bacterial structures (Supplementary Figure 7). However, Procrustes test analysis demonstrated that soil bacterial community compositions were not significantly correlated with ARG profiles on the basis of Bray–Curtis dissimilarity metrics (*R* = 0.3756, *p* > 0.05) (Figure 5).

### Fate of Manure-Derived Bacterial Communities and Antibiotic Resistance Genes in Soil

**FIGURE 6:**
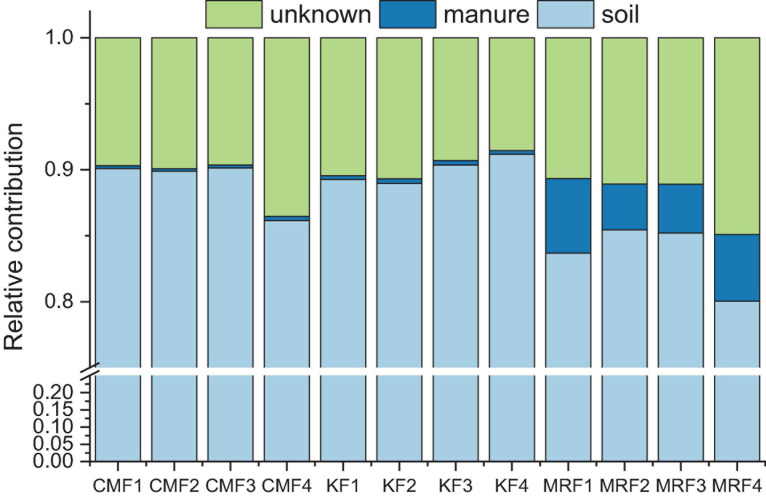
Relative contribution of the manure-derived, soil, and unknown sources of bacterial communities in the manure-treated soils estimated using FEAST analysis. KF, the combined application of NPK fertilizers and heat-treated chicken manure; CMF, the combined application of NPK fertilizers and chicken manure; MRF, the combined application of NPK fertilizers and mushroom residues; FEAST, fast expectation-maximization microbial source tracking. Data from unfertilized control (CK) served as the soil source.

Further, we employed FEAST to explore the fate of manure-derived bacteria in the soils. According to the analysis, manure of CM and K, respectively, exhibited 0.24 and 0.31% of the contributions for the bacterial communities in soils, while the average relative contribution of MR was 4.47% (Figure 6). The remaining proportions were due to “soil source” and “unknown source.” The indigenous bacterial communities in the sink were defined as “soil source,” and bacterial communities in CK were used as the “soil source.” Bacterial communities from MR survived more in soil than those from CM and K (Figure 6), which was consistent with the results of FEAST analysis for manure-derived ARGs (Table 2).

**TABLE 2:** Results of FEAST analysis showing the contribution of corresponding manure to ARGs in the soils.

	**Sink**	**Soil source**	**Manure source**	**Unknown**
F as the soil source	MRF	96.4 ± 4.1%	3.6 ± 4.1%	0.00 ± 0.0%
	KF	99.6 ± 0.2%	0.3 ± 0.2%	0.04 ± 0.1%
	CMF	99.6 ± 0.2%	0.4 ± 0.2%	0.00 ± 0.0%
CK as the soil source	MRF	95.8 ± 5.6%	4.2 ± 5.6%	0.00 ± 0.0%
	KF	99.7 ± 0.1%	0.2 ± 0.1%	0.03 ± 0.1%
	CMF	99.6 ± 0.1%	0.4 ± 0.2%	0.00 ± 0.0%

*FEAST, fast expectation-maximization microbial source tracking; ARGs, antibiotic resistance genes; MRF, the combined application of NPK fertilizers and mushroom residues; KF, the combined application of NPK fertilizers and heat-treated chicken manure; CMF, the combined application of NPK fertilizers and chicken manure.*

## Discussion

### Chicken Manure Had Different Antibiotic Resistance Gene Profiles With Mushroom Residues and Heat-Treated Chicken Manure

In this study, 330 ARG subtypes were quantified in manure by using HT-qPCR, and the results revealed that the detected ARG subtypes and the total ARG relative abundance were both much higher in chicken manure than in mushroom residues (Figure 1). Evolution and enrichment of ARGs are primarily stimulated by antibiotics (Pruden et al., 2006). Veterinary antibiotics have been widely used in livestock production for the prevention of diseases or as growth promoters (Zhang et al., 2015). ARB and ARGs may accumulate during the overuse of antibiotics in intestines of livestock. Therefore, it is not difficult to understand that ARGs in chicken manure were much more abundant than those in mushroom residue.

High temperatures (reach up to 90°C) during the fermentation process have been found to significantly enhance the removal of ARGs and MGEs from sewage sludge (Liao et al., 2018). As revealed by HT-qPCR or by RT-qPCR in this study, heat treatment (up to 260°C) can effectively remove 50 subtypes of ARGs from chicken manure and significantly reduce the total relative abundance of ARGs (Figure 1). Although ARGs and MGEs were still detected in the heat-treated chicken manure, most of them may exist in dead bacterial cells. Zhou et al. (2019) found that turning pig manure into biochar could noticeably reduce the level of the antibiotic resistome in soils compared with compost amendment. Although the sterilization effect of heat treatment at 260°C for 4 h may not be as thorough as that of turning it into biochar, the results of this study showed that compared with the direct application of fresh chicken manure, heat treatment of the manure also can reduce the detectable subtypes and relative abundance of ARGs in soils.

### Different Manures did Not Significantly Change the Antibiotic Resistance Gene Profiles in Alkaline Soil

Antibiotic deactivation and efflux pump were found to be the two major mechanisms for antibiotic resistance in the soils applied with different manures for 3 years (Supplementary Figure 1). Previous studies have reported the same phenomenon in sewage sludge-applied soils (Chen et al., 2016), reclaimed water-applied soils (Wang et al., 2014), swine farms (Zhu et al., 2013), and chicken manure or cattle manure-applied soils (Wang et al., 2020a). Efflux pumps are major players in both intrinsic and acquired resistance to drugs currently in use for the treatment of infectious diseases (Hernando-Amado et al., 2016), which are either chromosomal or plasmid-encoded (Rocha-Granados et al., 2020), and deserves more attention because they are often associated with the multidrug antibiotic resistance (Wang et al., 2020a).

Common agricultural practices like fertilization and irrigation are known to affect soil resistome. Many studies have found that long-term manure application significantly increased the diversity and abundances of ARGs and MGEs (Peng et al., 2017; Han et al., 2018). However, in some studies, such significant trends of ARGs caused by long-term application of manure could not be observed (Tang et al., 2015; Wang et al., 2018). According to HT-qPCR, the distribution and total relative abundance of ARGs among manured soils had no significant difference in this study (Figure 2, Supplementary Figure 3, and Supplementary Table 1); soil types (Wang et al., 2020a), sources of animal manure (Zhang et al., 2017), tillage and cropping systems (Wang et al., 2018), and time (Liu et al., 2017) and application frequency (Heuer et al., 2011) may lead to the inconsistent results. In addition, a subset of ARGs analyzed in these studies may be one of the reasons; different from the result of HT-qPCR, the total relative abundance of 13 ARGs quantified by RT-qPCR was significantly higher in CMF soils (Figure 2). Therefore, more types of ARGs considered may provide a more comprehensive insight into the influence of manure fertilization in an agricultural environment. The different findings detected in HT-qPCR and RT-qPCR may result from the differences in primer sequences, polymerase, qPCR volume (100 nl vs. 20 μl), and number of primer sets (one pair in RT-qPCR vs. multiple primer pairs for some genes).

Antibiotic resistance genes profiles in manured soils were not significantly changed, yet among the four fertilized treatments, CMF soils had more detected ARG subtypes and higher ARG abundance level (Figure 2). Although differences were not significant, they still indicated that application of fresh chicken manure induced some ARG subtypes in soil. Previous studies found that application of chicken manure generally increased the abundance of some ARGs (Urra et al., 2019; Liu et al., 2021). This study further verified the result with long-term applications in the field. The soil in this study was a sandy loam (alluvial-aquic) soil with alkaline characteristics. Our previous study had found that *tetL* in this alkaline soil was enriched after long-term fertilized with plant-derived organic manure, and survival of the *Firmicutes* bacteria that carried *tetL* may be the cause (Peng et al., 2018). In this study, compared with F, the relative abundance of *tetL* quantified by RT-qPCR was also increased in different manure-amended soils (Supplementary Figure 5), which may be due to the same reason as our previous finding.

### Small Proportion of Manure-Derived Bacteria and Antibiotic Resistance Gene Survival in Soil May Be the Main Reason for Its Minor Effects on Soil Antibiotic Resistance Gene Abundance

The succession of microbial community structure is the main factor that influenced the variation of ARGs during sewage sludge composting (Su et al., 2015), anaerobic digestion of dairy manure (Sun et al., 2016), and manure composting (Yin et al., 2017; Awasthi et al., 2019). Changes of soil resistome induced by manure application mainly resulted from the alteration of soil bacteria communities rather than the horizontal gene transfer (Liu et al., 2017). In this study, bacterial communities were significantly changed by different manure treatments (Supplementary Figure 6 and Supplementary Table 2), while manure application only showed some minor effects on ARG profile (Figure 3, Supplementary Figure 3, and Supplementary Table 1), and bacteria community compositions were not significantly correlated with ARG profiles (Figure 5). These results suggested that the variation of ARG profile is not the inevitable result of bacterial community change in manure-treated soil. As mentioned above, manure application did not always lead to significant increase of ARG abundance in soil, and the reasons for this phenomenon are very complex. Finding out the reasons may be of great value for guiding us to take effective measures to reduce ARG diffusion in farmland.

Fast expectation maximization microbial source tracking and SourceTracker were commonly used machine-learning classification tools in microbial source tracking, which were used in manure-associated community analysis (Gou et al., 2018) or quantifying the contributions of potential sources to ARGs in the river sediments (Chen et al., 2020). As a novel microbial source tracking method, FEAST has some distinct advantages on its estimation accuracy particularly if the sink community contains species/gene from an uncharacterized source (Shenhav et al., 2019; Chen et al., 2020). Here, FEAST was used to apportion the contributions of soil, mushroom residues, and chicken manure on the presence of bacteria communities and ARGs in the manure-amended soils, and results showed that MR-derived bacteria and ARGs survived more than those derived from CM and K (Figure 6 and Table 2). The formation environment of mushroom residues is more similar to that of the soil environment than chicken gut microenvironment. In addition, soil microbial diversity is the key factor controlling the extent to which bacterial invaders can be established (van Elsas et al., 2012; Peng et al., 2016). Thus, the indigenous soil microbial community may also play an important role in inhibiting the CM- and K-derived bacteria survival in soil.

The total relative abundance of ARGs in CMF soils was 1.41 times higher than that of MRF and KF soils even not significantly (*p* > 0.05), and three ARGs were significantly enriched (Figures 2, 3). We speculated that manure affect soil ARGs from three ways: firstly, nutrients carried by manure cause the proliferation of endogenous bacteria carrying by ARGs; secondly, ARGs from manure transfer to soil endogenous microorganisms; and finally, manure-derived ARG survival in soil. Nutrients can enhance the effect of selective pressures of ARGs (Deng et al., 2020); even sterilized manure also can result in a little increase in the relative abundance of tetracycline-resistant bacteria in soil (Peng et al., 2016). Therefore, the seven significantly enriched ARGs in three manure treatments (Figure 3) may result from the proliferation of endogenous resistance using nutrients carried by the manures. Bacterial abundances in manure-amended soils were not significantly increased (Figure 2D); this may result from the fertilization treatments in this study being loaded with equal nitrogen into the soils. From the perspective of MGEs, the total relative abundance of MGEs was not significantly different among three manure-treated soils (Figure 2), connections between ARGs and MGEs were not improved by manure application (Supplementary Figure 4). It has been demonstrated that MGEs could potentially enhance the accumulation and persistence of ARGs in manure/sludge-amended soils (Gaze et al., 2011). However, horizontal gene transfer occurrence of ARGs via MGEs may depend, at least partly, on its types; the limited distribution of some genes is possibly associated with their limited host range (Chen et al., 2016). Therefore, horizontal gene transfer via MGEs may not be the main reason leading to the minor increase of the total ARG abundance in CMF soils. Although CM-derived bacteria contributed a little proportion (0.19–0.31%) to the CMF soil bacteria community (Figure 5), the relative abundance of ARGs in CM was higher than that of MR and K (Figure 1). Thus, when the same proportion of bacteria survived in soil, CM-derived bacteria may have a higher impact on soil ARGs than that from MR and K. FEAST analysis showed that CM-derived ARGs contributed 0.4% to the ARGs in CMF soils, while K-derived ARGs contributed 0.3% (Table 2), and the enriched ARGs in CMF soils may be from the surplus 0.1% contribution of CM. MR carried a small amount of ARGs (Figure 1), so it did not significantly affect soil ARGs after being applied for 3 years, even though MR-derived ARGs contributed 3.6% to the soil ARGs (Table 2).

The detected ARG subtypes were significantly decreased, and the total abundance of ARGs was reduced by 29.12% in KF soils compared with that in CMF soils (Figure 2). This indicated that heat treatment of chicken manure may efficiently reduce the amount of ARGs introduced into soil, thus making its abundance level similar to that of MRF soils. This result further verified our early findings in mesocosm and greenhouse experiments that nutrients and tetracycline resistance genes not carried by live bacteria in sterilized manure contribute little to the accumulation of tetracycline-resistant bacteria and genes in soils (Peng et al., 2016). The total abundance of MGEs was also the lowest in KF soils (Figure 2), which inferred that the opportunities of ARG transfer to other species may also be reduced. In addition, after the oil bath kettle was started, the high temperature of the oil bath kettle can be maintained for a period of time after the temperature is raised, and the inner part of the equipment is closed, leaving only the steam outlet. Therefore, the energy consumption is relatively low: it needs about 48-kW electric energy to treat 1 ton of chicken manure. Whether the application of heat-treated manure can decrease the migration of ARGs in other types of soil and the removal efficiency of this oil bath high-temperature treatment kettle on ARGs from other animal manure needs further study.

## Conclusion

In this study, 3 years’ application of mushroom residue, chicken manure, and heat-treated chicken manure significantly changed the soil bacteria community compositions. However, the profiles and abundance of the ARGs in the fertilized soils were not significantly changed but only showed some minor increase in chicken manure-amended soils. In quantitative apportionment, the analysis of FEAST demonstrated that intrinsic ARGs from soil dominated 95.8–99.7% of the relative contribution of ARGs in the manure-amend soils. The contribution of chicken manure was similar to that of heat-treated manure (0.4 and 0.2%), and the contribution of both was lower than that of mushroom residues (4.2%). Although the application of the fresh chicken manure for three consecutive years had no significant effect on soil ARGs, the total abundance of ARGs had increased slightly. Heat treatment obviously reduced the total abundance of ARGs and MGEs in chicken manure, and the total ARG abundance in the heat-treated manure-amended soils was similar to that observed in the soils applied with mushroom residue. It suggests that treating animal manure with high temperature may be an environmentally sustainable technology to reduce the public health risk of ARGs derived from intensive animal farming.

## Data Availability Statement

The data presented in the study are deposited in the NCBI sequence read archive (SRA) repository, accession number (PRJNA669308).

## Author Contributions

XL and RC initiated the project. RC and YW supplied soil materials and reagents. YW contributed his scientific advice during the work. SP performed the experiments, analyzed the data, and wrote the manuscript. All authors reviewed the manuscript.

## Conflict of Interest

The authors declare that the research was conducted in the absence of any commercial or financial relationships that could be construed as a potential conflict of interest.

## Footnotes

1 https://github.com/cozygene/FEAST

## References

[B1] Awasthi M. K. Liu T. Chen H. Verma S. Duan Y. Awasthi S. K. (2019). The behavior of antibiotic resistance genes and their associations with bacterial community during poultry manure composting. *Bioresour. Technol.* 280 70–78. 10.1016/j.biortech.2019.02.030 30754007

[B2] Chen H. Li Y. Sun W. Song L. Zuo R. Teng Y. (2020). Characterization and source identification of antibiotic resistance genes in the sediments of an interconnected river-lake system. *Environ. Int.* 137:105538. 10.1016/j.envint.2020.105538 32028174

[B3] Chen Q. An X. Li H. Su J. Ma Y. Zhu Y. G. (2016). Long-term field application of sewage sludge increases the abundance of antibiotic resistance genes in soil. *Environ. Int.* 92-93 1–10. 10.1016/j.envint.2016.03.026 27043971

[B4] Deng W. Zhang A. Chen S. He X. Jin L. Yu X. (2020). Heavy metals, antibiotics and nutrients affect the bacterial community and resistance genes in chicken manure composting and fertilized soil. *J. Environ. Manage* 257:109980. 10.1016/j.jenvman.2019.109980 31868641

[B5] Edgar R. C. Haas B. J. Clemente J. C. Quince C. Knight R. (2011). UCHIME improves sensitivity and speed of chimeradetection. *Bioinformatics* 27 2194–2200. 10.1093/bioinformatics/btr381 21700674 PMC3150044

[B6] Evanylo G. Sherony C. Spargo J. Starner D. Brosius M. Haering K. (2008). Soil and water environmental effects of fertilizer-, manure-, and compost based fertility practices in an organic vegetable cropping system. *Agric. Ecosyst. Environ.* 127 50–58. 10.1016/j.agee.2008.02.014

[B7] Feng Y. Chen R. Hu J. Zhao F. Wang J. Chu H. (2015). *Bacillus asahii* comes to the fore in organic manure fertilized alkaline soils. *Soil Biol. Biochem.* 81 186–194. 10.1016/j.soilbio.2014.11.021

[B8] Forsberg K. J. Patel S. Gibson M. K. Lauber C. L. Knight R. Fierer N. (2014). Bacterial phylogeny structures soil resistomes across habitats. *Nature* 509 612–616. 10.1038/nature13377 24847883 PMC4079543

[B9] Forsberg K. J. Reyes A. Wang B. Selleck E. M. Sommer M. O. Dantas G. (2012). The shared antibiotic resistome of soil bacteria and human pathogens. *Science* 337 1107–1111. 10.1126/science.1220761 22936781 PMC4070369

[B10] Gaze W. H. Zhang L. H. Abdouslam N. A. Hawkey P. M. Calvo-Bado L. Royle J. (2011). Impacts of anthropogenic activity on the ecology of class 1 integrons and integron-associated genes in the environment. *ISME J.* 5 1253–1261. 10.1038/ismej.2011.15 21368907 PMC3146270

[B11] Gou M. Hu H. W. Zhang Y. J. Wang J. T. Hayden H. Tang Y. Q. (2018). Aerobic composting reduces antibiotic resistance genes in cattle manure and the resistome dissemination in agricultural soils. *Sci. Total Environ.* 612 1300–1310. 10.1016/j.scitotenv.2017.09.028 28898936

[B12] Guo X. P. Zhao S. Chen Y. R. Yang J. Hou L. J. Liu M. (2020). Antibiotic resistance genes in sediments of the Yangtze estuary: from 2007 to 2019. *Sci. Total Environ.* 744:140713. 10.1016/j.scitotenv.2020.140713 32693274

[B13] Han X. M. Hu H. W. Chen Q. L. Yang L. Y. Li H. L. Zhu Y. G. (2018). Antibiotic resistance genes and associated bacterial communities in agricultural soils amended with different sources of animal manures. *Soil Biol. Biochem.* 126 91–102. 10.1016/j.soilbio.2018.08.018

[B14] Hernando-Amado S. Blanco P. Alcalde-Rico M. Corona F. Reales-Calderon J. A. Sanchez M. B. (2016). Multidrug efflux pumps as main players in intrinsic and acquired resistance to antimicrobials. *Drug Resist. Updat.* 28 13–27. 10.1016/j.drup.2016.06.007 27620952

[B15] Heuer H. Solehati Q. Zimmerling U. Kleineidam K. Schloter M. Muller T. (2011). Accumulation of sulfonamide resistance genes in arable soils due to repeated application of manure containing sulfadiazine. *Appl. Environ. Microbiol.* 77 2527–2530. 10.1128/aem.02577-10 21296942 PMC3067416

[B16] Kang Y. Hao Y. Shen M. Zhao Q. Li Q. Hu J. (2016). Impacts of supplementing chemical fertilizers with organic fertilizers manufactured using pig manure as a substrate on the spread of tetracycline resistance genes in soil. *Ecotox. Environ. Safe* 130 279–288. 10.1016/j.ecoenv.2016.04.028 27152658

[B17] Lee K. Kim D. W. Lee D. H. Kim Y. S. Bu J. H. Cha J. H. (2020). Mobile resistome of human gut and pathogen drives anthropogenic bloom of antibiotic resistance. *Microbiome* 8 1–14. 31910889 10.1186/s40168-019-0774-7 PMC6947943

[B18] Levy S. B. Marshall B. (2004). Antibacterial resistance worldwide: causes, challenges and responses. *Nat. Med.* 10 (12 Suppl) S122–S129. 10.1038/nm1145 15577930

[B19] Li F. Kong Q. Zhang Q. Wang H. Wang L. Luo T. (2020). Spent mushroom substrates affect soil humus composition, microbial biomass and functional diversity in paddy fields. *Appl. Soil Ecol.* 149:103489. 10.1016/j.apsoil.2019.103489

[B20] Liao H. Lu X. Rensing C. Friman V. P. Geisen S. Chen Z. (2018). Hyperthermophilic composting accelerates the removal of antibiotic resistance genes and mobile genetic elements in sewage sludge. *Environ. Sci. Technol.* 52 266–276. 10.1021/acs.est.7b04483 29199822

[B21] Liu P. Jia S. He X. Zhang X. Ye L. (2017). Different impacts of manure and chemical fertilizers on bacterial community structure and antibiotic resistance genes in arable soils. *Chemosphere* 188 455–464. 10.1016/j.chemosphere.2017.08.162 28898777

[B22] Liu W. Ling N. Guo J. Ruan Y. Wang M. Shen Q. (2021). Dynamics of the antibiotic resistome in agricultural soils amended with different sources of animal manures over three consecutive years. *J. Hazard Mater.* 401:123399. 10.1016/j.jhazmat.2020.123399 32763695

[B23] Martin M. (2011). Cutadapt removes adapter sequences from high-throughput sequencing reads. *EMBnet J.* 17 10–12. 10.14806/ej.17.1.200

[B24] McInnes R. S. McCallum G. E. Lamberte L. E. van Schaik W. (2020). Horizontal transfer of antibiotic resistance genes in the human gut microbiome. *Curr. Opin. Microbiol.* 53 35–43. 10.1016/j.mib.2020.02.002 32143027

[B25] Muurinen J. Stedtfeld R. Karkman A. Parnanen K. Tiedje J. Virta M. (2017). Influence of manure application on the environmental resistome under finnish agricultural practice with restricted antibiotic use. *Environ. Sci. Technol.* 51 5989–5999. 10.1021/acs.est.7b00551 28453251

[B26] Nesme J. Simonet P. (2015). The soil resistome: a critical review on antibiotic resistance origins, ecology and dissemination potential in telluric bacteria. *Environ. Microbiol.* 17 913–930. 10.1111/1462-2920.12631 25286745

[B27] Peng S. Dolfing J. Feng Y. Wang Y. Lin X. (2018). Enrichment of the Antibiotic Resistance Gene tet(L) in an alkaline soil fertilized with plant derived organic manure. *Front. Microbiol.* 9:1140. 10.3389/fmicb.2018.01140 29904377 PMC5990627

[B28] Peng S. Feng Y. Wang Y. Guo X. Chu H. Lin X. (2017). Prevalence of antibiotic resistance genes in soils after continually applied with different manure for 30 years. *J. Hazard Mater.* 340 16–25. 10.1016/j.jhazmat.2017.06.059 28711829

[B29] Peng S. Wang Y. Zhou B. Lin X. (2015). Long-term application of fresh and composted manure increase tetracycline resistance in the arable soil of eastern China. *Sci. Total Environ.* 506 279–286. 10.1016/j.scitotenv.2014.11.010 25460961

[B30] Peng S. Zhou B. Wang Y. Lin X. Wang H. Qiu C. (2016). Bacteria play a more important role than nutrients in the accumulation of tetracycline resistance in manure-treated soil. *Biol. Fertil. Soils* 52 655–663. 10.1007/s00374-016-1105-9

[B31] Pruden A. Pei R. Storteboom H. Carlson K. H. (2006). Antibiotic resistance genes as emerging contaminants: studies in northern Colorado. *Environ. Sci. Technol.* 40 7445–7450. 10.1021/es060413l 17181002

[B32] Qian X. Gu J. Sun W. Wang X. J. Su J. Q. Stedfeld R. (2017). Diversity, abundance, and persistence of antibiotic resistance genes in various types of animal manure following industrial composting. *J. Hazard. Mater.* 344:716. 10.1016/j.jhazmat.2017.11.020 29154097

[B33] Rocha-Granados M. C. Zenick B. Englander H. E. Mok W. W. K. (2020). The social network: Impact of host and microbial interactions on bacterial antibiotic tolerance and persistence. *Cell. Signal.* 75:109750. 10.1016/j.cellsig.2020.109750 32846197

[B34] Shenhav L. Thompson M. Joseph T. A. Briscoe L. Furman O. Bogumil D. (2019). FEAST: fast expectation-maximization for microbial source tracking. *Nat. Methods* 16 627–632. 10.1038/s41592-019-0431-x 31182859 PMC8535041

[B35] Su J. Q. Wei B. Ou-Yang W. Y. Huang F. Y. Zhao Y. Xu H. J. (2015). Antibiotic resistome and its association with bacterial communities during sewage sludge composting. *Environ. Sci. Technol.* 49 7356–7363. 10.1021/acs.est.5b01012 26018772

[B36] Sun W. Qian X. Gu J. Wang X. J. Duan M. L. (2016). Mechanism and effect of temperature on variations in antibiotic resistance genes during anaerobic digestion of dairy manure. *Sci. Rep.* 6:30237. 27444518 10.1038/srep30237 PMC4957233

[B37] Sun Y. Qiu T. Gao M. Shi M. Zhang H. Wang X. (2019). Inorganic and organic fertilizers application enhanced antibiotic resistome in greenhouse soils growing vegetables. *Ecotoxicol. Environ. Saf.* 179 24–30. 10.1016/j.ecoenv.2019.04.039 31022652

[B38] Tang X. Lou C. Wang S. Lu Y. Liu M. Hashmi M. Z. (2015). Effects of long-term manure applications on the occurrence of antibiotics and antibiotic resistance genes (ARGs) in paddy soils: evidence from four field experiments in south of China. *Soil Biol. Biochem.* 90 179–187. 10.1016/j.soilbio.2015.07.027

[B39] Urra J. Alkorta I. Lanzén A. Mijangos I. Garbisu C. (2019). The application of fresh and composted horse and chicken manure affects soil quality, microbial composition and antibiotic resistance. *Appl. Soil Ecol.* 135 73–84. 10.1016/j.apsoil.2018.11.005

[B40] van Elsas J. D. Chiurazzi M. Mallon C. A. Elhottova D. Kristufek V. Salles J. F. (2012). Microbial diversity determines the invasion of soil by a bacterial pathogen. *Proc. Nat. Acad. Sci. U.S.A.* 109 1159–1164. 10.1073/pnas.1109326109 22232669 PMC3268289

[B41] Wang F. Xu M. Stedtfeld R. D. Sheng H. Fan J. Liu M. (2018). Long-term effect of different fertilization and cropping systems on the soil antibiotic resistome. *Environ. Sci. Technol.* 52 13037–13046. 10.1021/acs.est.8b04330 30375866

[B42] Wang F. H. Qiao M. Su J. Q. Chen Z. Zhou X. Zhu Y. G. (2014). High throughput profiling of antibiotic resistance genes in urban park soils with reclaimed water irrigation. *Environ. Sci. Technol.* 48 9079–9085. 10.1021/es502615e 25057898

[B43] Wang L. Wang J. Wang J. Zhu L. Conkle J. L. Yang R. (2020a). Soil types influence the characteristic of antibiotic resistance genes in greenhouse soil with long-term manure application. *J. Hazard Mater.* 392:122334. 10.1016/j.jhazmat.2020.122334 32092657

[B44] Wang X. Yang Y. Zhao J. Nie J. Zang H. Zeng Z. (2020b). Yield benefits from replacing chemical fertilizers with manure under water deficient conditions of the winter wheat – summer maize system in the North China Plain. *Eur. J. Agron.* 119:126118. 10.1016/j.eja.2020.126118

[B45] Wu X. Tian Z. Lv Z. Chen Z. Liu Y. Yong X. (2020). Effects of copper salts on performance, antibiotic resistance genes, and microbial community during thermophilic anaerobic digestion of swine manure. *Bioresour. Technol.* 300:122728. 10.1016/j.biortech.2019.122728 31926471

[B46] Xiong W. Sun Y. Zhang T. Ding X. Li Y. Wang M. (2015). Antibiotics, antibiotic resistance genes, and bacterial community composition in fresh water aquaculture environment in China. *Microb. Ecol.* 70 425–432. 10.1007/s00248-015-0583-x 25753824

[B47] Yao T. Chen R. Zhang J. Feng Y. Huang M. Lin X. (2020). Divergent patterns of microbial community composition shift under two fertilization regimes revealed by responding species. *Appl. Soil Ecol.* 154:103590. 10.1016/j.apsoil.2020.103590

[B48] Yi X. Yu L. Chang S.-H.-E. Yin C. Wang H. Zhang Z. (2021). The effects of China’s Organic-Substitute-Chemical-Fertilizer (OSCF) policy on greenhouse vegetable farmers. *J. Cleaner Prod.* 297:126677. 10.1016/j.jclepro.2021.126677

[B49] Yin Y. Gu J. Wang X. Song W. Zhang K. Sun W. (2017). Effects of copper addition on copper resistance, antibiotic resistance genes, and intl1 during swine manure composting. *Front. Microbiol.* 8:344. 10.3389/fmicb.2017.00344 28316595 PMC5335643

[B50] Zhang Q. Q. Ying G. G. Pan C. G. Liu Y. S. Zhao J. L. (2015). Comprehensive evaluation of antibiotics emission and fate in the river basins of China: source analysis, multimedia modeling, and linkage to bacterial resistance. *Environ. Sci. Technol.* 49 6772–6782. 10.1021/acs.est.5b00729 25961663

[B51] Zhang T. Zhang X. X. Ye L. (2011). Plasmid metagenome reveals high levels of antibiotic resistance genes and mobile genetic elements in activated sludge. *PLoS One* 6:e26041. 10.1371/journal.pone.0026041 22016806 PMC3189950

[B52] Zhang Y. J. Hu H. W. Chen Q. L. Singh B. K. Yan H. Chen D. (2019). Transfer of antibiotic resistance from manure-amended soils to vegetable microbiomes. *Environ. Int.* 130:104912. 10.1016/j.envint.2019.104912 31220751

[B53] Zhang Y. J. Hu H. W. Gou M. Wang J. T. Chen D. He J. Z. (2017). Temporal succession of soil antibiotic resistance genes following application of swine, cattle and poultry manures spiked with or without antibiotics. *Environ. Pollut.* 231 (Pt 2) 1621–1632. 10.1016/j.envpol.2017.09.074 28964602

[B54] Zhou X. Qiao M. Su J. Q. Wang Y. Cao Z. H. Cheng W. D. (2019). Turning pig manure into biochar can effectively mitigate antibiotic resistance genes as organic fertilizer. *Sci. Total Environ.* 649 902–908. 10.1016/j.scitotenv.2018.08.368 30179818

[B55] Zhu Y. G. Johnson T. A. Su J. Q. Qiao M. Guo G. X. Stedtfeld R. D. (2013). Diverse and abundant antibiotic resistance genes in Chinese swine farms. *Proc. Natl. Acad. Sci. U.S.A.* 110 3435–3440. 10.1073/pnas.1222743110 23401528 PMC3587239

