# Supplementary material for: Chicken Manure and Mushroom Residues Affect Soil Bacterial Community Structure but Not the Bacterial Resistome When Applied at the Same Rate of Nitrogen for 3 Years
Source: Front Microbiol. 2021 May 21;12:618693. doi: 10.3389/fmicb.2021.618693 (PMC8177108; doi:10.3389/fmicb.2021.618693)
Supplement: Supplementary file 1 [file Data_Sheet_1.docx]

**Supporting Information**

**for**

**Chicken manure and mushroom residues affect soil bacterial community structure but not the bacterial resistome when applied at the same rate of nitrogen for 3 years**

Shuang Peng ^a,b^, Yiming Wang ^b,c*^, Ruirui Chen ^b,c*^, Xiangui Lin ^b^

^a^ College of Environment and Ecology, Jiangsu Open University, Nanjing, Jiangsu 210017, China

^b^ State Key Laboratory of Soil and Sustainable Agriculture, Institute of Soil Science, Chinese Academy of Sciences, Nanjing, Jiangsu 210008, China

^c^ Jiangsu Collaborative Innovation Center for Solid Organic Waste Resource Utilization, Nanjing 210095, Jiangsu, China

*Corresponding author, Mailing address: Department of Biology and Biochemistry, Institute of Soil Science, Chinese Academy of Sciences, Beijing East Road, 71 Nanjing 210008, PR China.

Tel.: (+086) 02586881337. Fax:(+086) 02586881000.

e-mail address: [ymwang@issas.ac.cn](mailto:ymwang@issas.ac.cn); rrchen@issas.ac.cn

**Fig. S1**. ARGs detected in each treatment were classified based on the mechanism of resistance. CK, unfertilized control; F, NPK fertilizers; KF, the combined application of NPK fertilizers and heat treated chicken manure; CMF, the combined application of NPK fertilizers and chicken manure; MRF, the combined application of NPK fertilizers and mushroom residues.


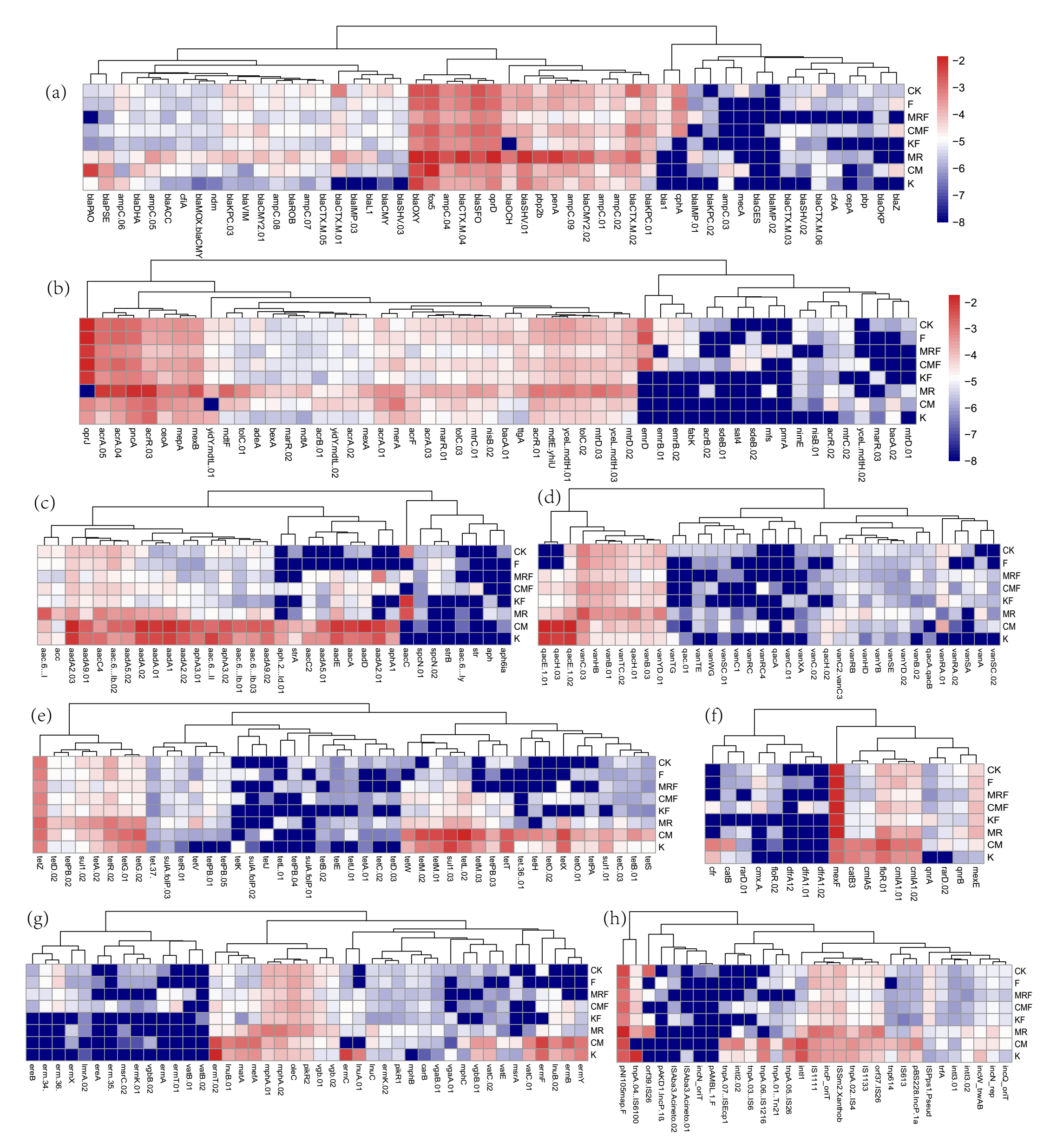


**Fig. S2**. The relative abundance of ARGs and MGEs in soils with different treatment. The color intensity in each panel shows the average relative abundance (log scaled) of each ARGs or MGEs in four samples. Lower case letters indicated ARGs belong to beta-lactam (a), multidrug other (b), aminoglycoside (c), vancomycin and disinfectant (d), tetracycline and sulfonamide (e), phenicols and trimethoprim (f), MLSB (g) and MGEs (h).


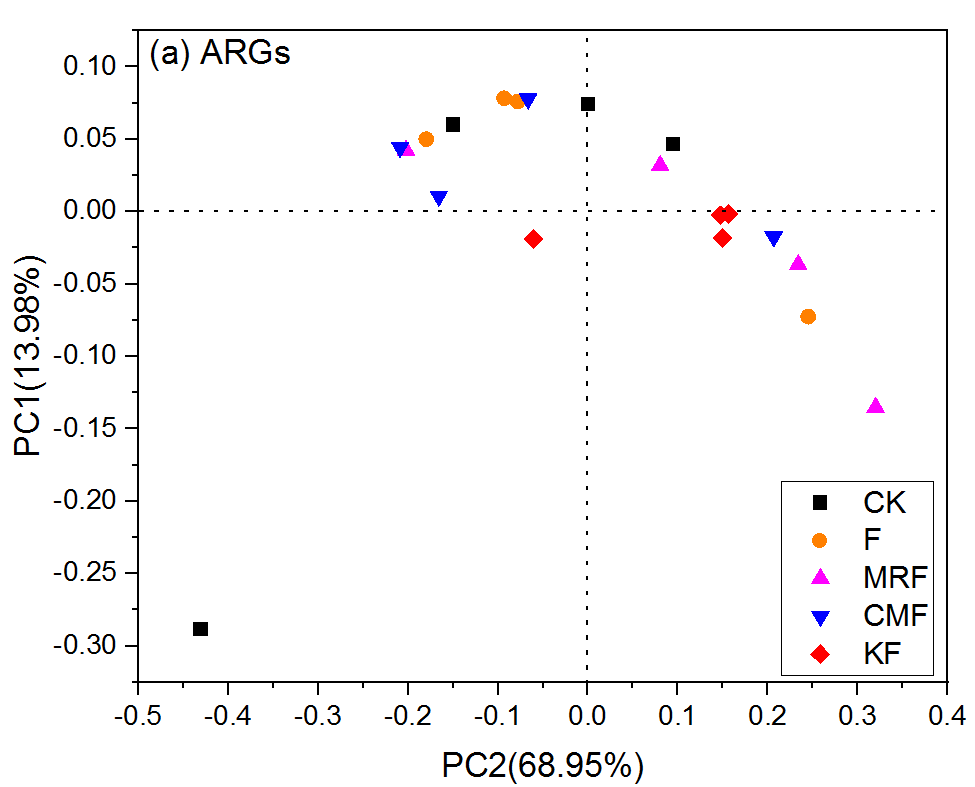

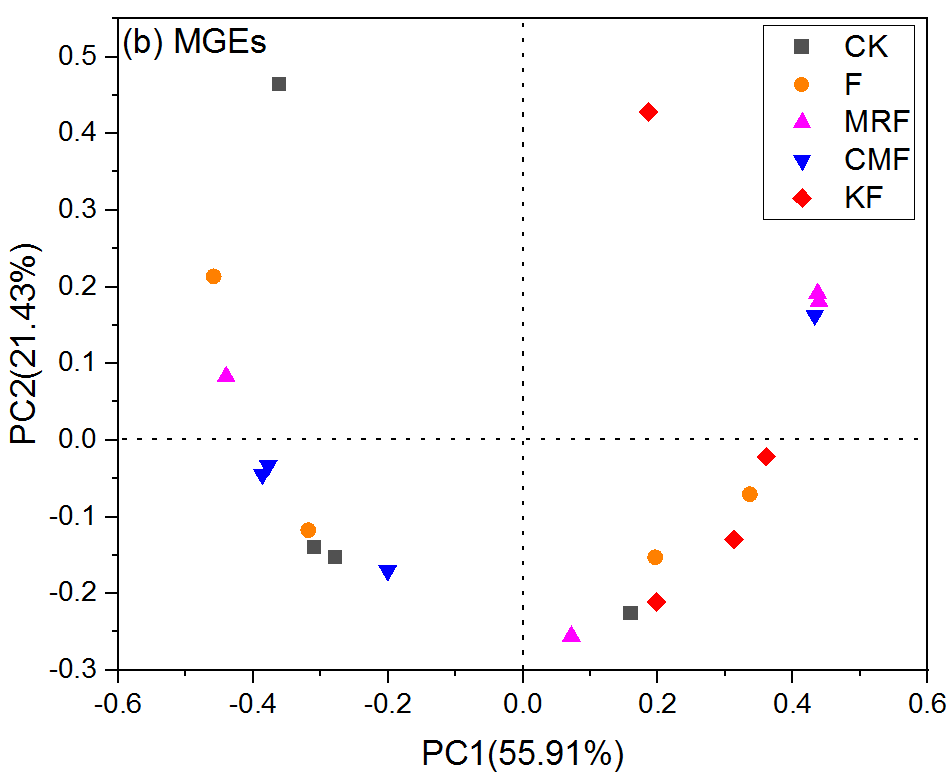


**Fig. S3**. Principal coordinate analysis (PCoA) based on the Bray-Curtis distance showing the overall distribution patterns of ARGs (a) and MGEs (b) in different manure amended soils. CK, unfertilized control; F, NPK fertilizers; KF, the combined application of NPK fertilizers and heat treated chicken manure; CMF, the combined application of NPK fertilizers and chicken manure; MRF, the combined application of NPK fertilizers and mushroom residues.


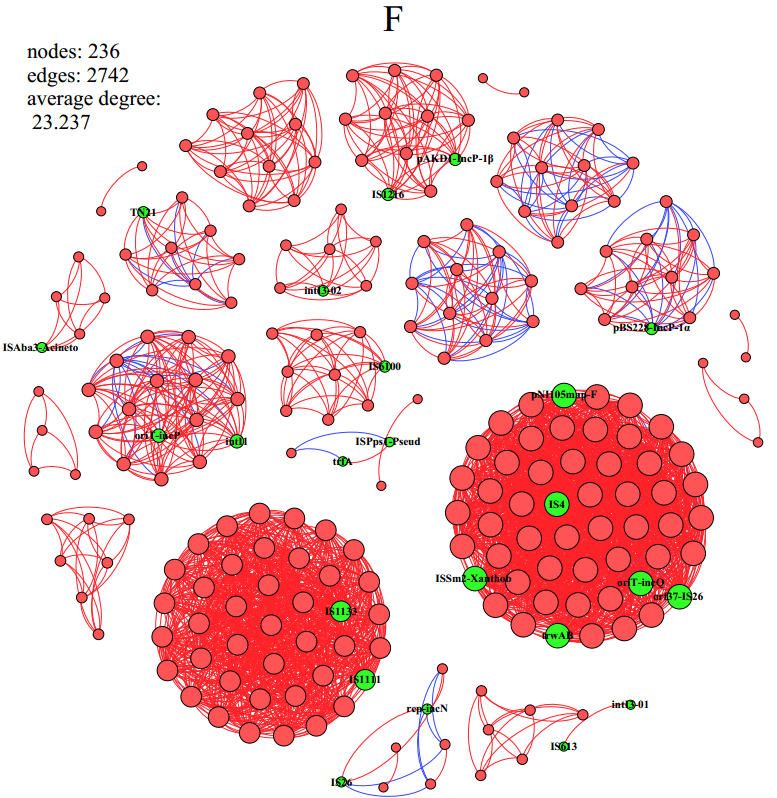

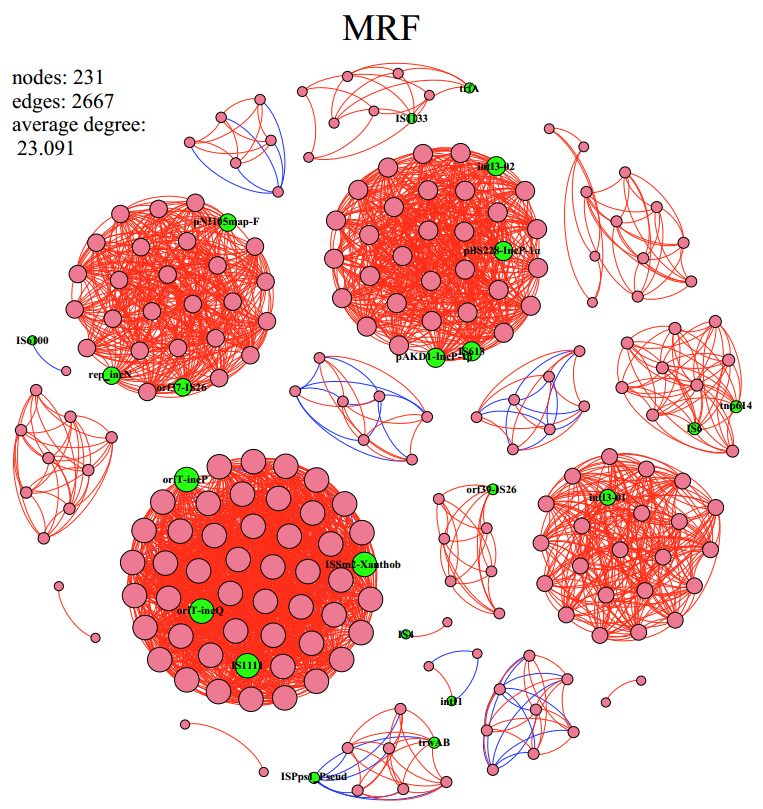

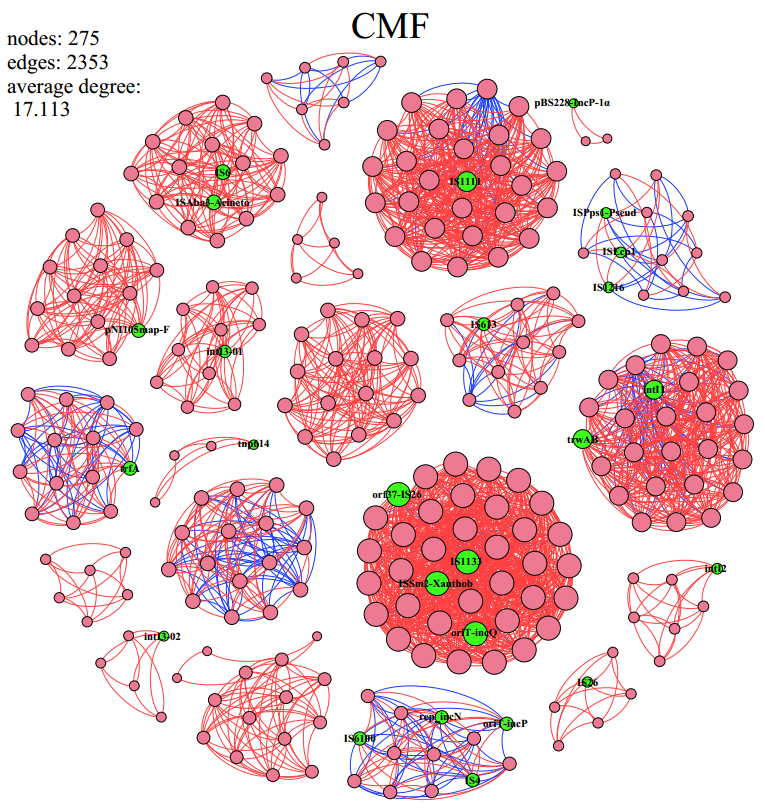

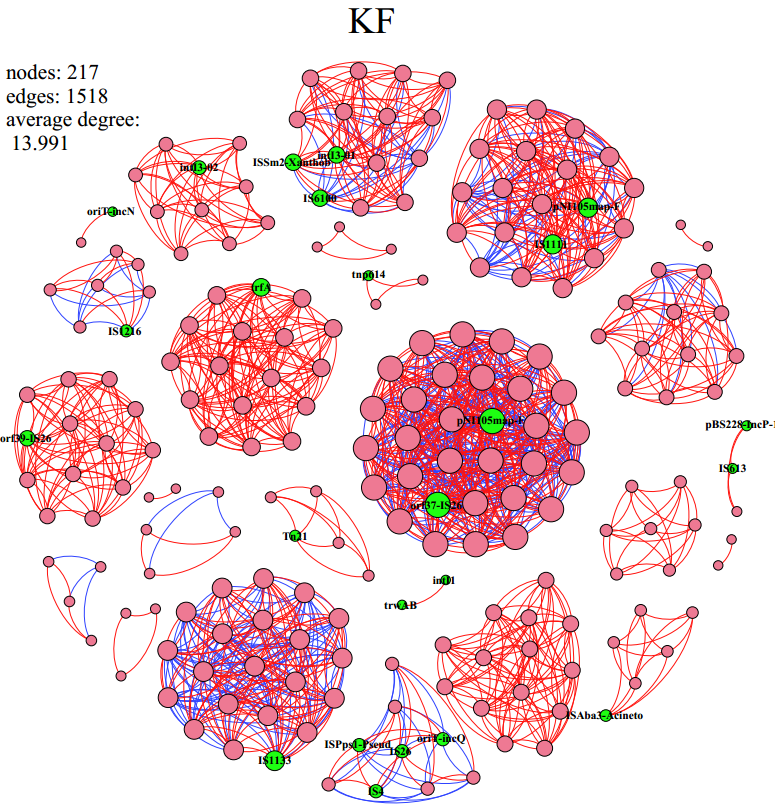


**Fig. S4.** The network analysis showing the co-occurrence patterns among the detected ARG and MGE subtypes in the soils. The nodes with red or green colors respectively represent ARGs and MGEs. A connection represents a strong (Spearman's correlation coefficient R^2^ > 0.9) and significant (*P*-value < 0.01) correlation. Edges’ color (red and blue) respectively represent positive and negative correlations and node size weighted according to the number of significant correlations between nodes. The size of each node is proportional to the number of connections. F, NPK fertilizers; KF, the combined application of NPK fertilizers and heat treated chicken manure; CMF, the combined application of NPK fertilizers and chicken manure; MRF, the combined application of NPK fertilizers and mushroom residues.

**Fig. S5.** The average relative abundance of the ARGs in soil samples quantified by RT-qPCR, the different lowercase letters indicate statistically significant differences at *p* < 0.05. CK, unfertilized control; F, NPK fertilizers; KF, the combined application of NPK fertilizers and heat treated chicken manure; CMF, the combined application of NPK fertilizers and chicken manure; MRF, the combined application of NPK fertilizers and mushroom residues.

**Fig. S6.** Non-metric multidimensional scaling (NMDS) ordination of bacterial communities separated by manure treatments. Bonferroni-corrected p-values were applied in all treatments with ANOSIM. (R, the degree of separation between test groups ranging from −1 to 1; R= 0, not different; R=1, completely different; p-values were based on 999 permutations). CK, unfertilized control; F, NPK fertilizers; KF, the combined application of NPK fertilizers and heat treated chicken manure; CMF, the combined application of NPK fertilizers and chicken manure; MRF, the combined application of NPK fertilizers and mushroom residues.


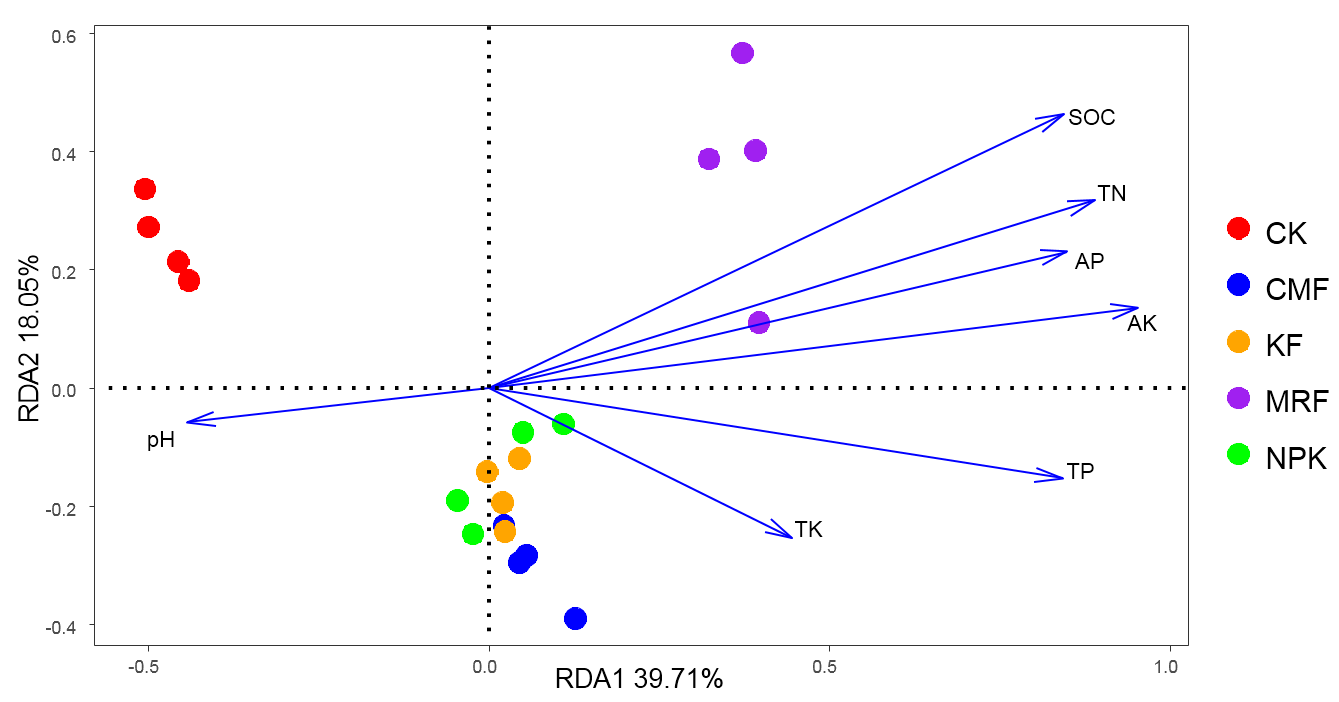


**Fig. S7.** Redundancy analysis (RDA) of the relationship between soil chemical characteristics and bacterial communities. Soil factors showed in blue arrow include AK (available potassium), AP (available phosphorus), pH, TN (total nitrogen), TP (total phosphorus), TK (total potassium) and SOC (Soil organic carbon). CK, unfertilized control; F, NPK fertilizers; KF, the combined application of NPK fertilizers and heat treated chicken manure; CMF, the combined application of NPK fertilizers and chicken manure; MRF, the combined application of NPK fertilizers and mushroom residues.

**Table S1** ANOSIM pairwise comparisons of ARGs and MGEs compositions for the different fertilization treatments.

|  | ARGs | | MGEs | |
| --- | --- | --- | --- | --- |
| Comparison | R statistic | *p*-value | R statistic | *p*-value |
| CK vs F | -0.031 | 0.551 | -0.156 | 0.776 |
| CK vs MRF | -0.010 | 0.462 | 0.021 | 0.407 |
| CK vs CMF | -0.115 | 0.922 | -0.115 | 0.812 |
| CK vs KF | 0.177 | 0.143 | 0.302 | 0.049 |
| F vs MRF | 0.073 | 0.292 | -0.052 | 0.508 |
| F vs CMF | -0.083 | 0.607 | -0.115 | 0.833 |
| F vs KF | 0.156 | 0.158 | -0.010 | 0.522 |
| MRF vs CMF | 0.010 | 0.305 | -0.021 | 0.274 |
| MRF vs KF | -0.042 | 0.548 | -0.031 | 0.587 |
| CMF vs KF | 0.208 | 0.227 | 0.198 | 0.134 |

R statistics represent difference of mean ranks between the two groups. Values closer to 0 indicates not different between the two groups compared, *p*-values were based on 999 permutations.

**Table S2** ANOSIM and PERMANONA pairwise comparisons of bacterial community compositions for the different fertilization treatments.

|  | ANOSIM | | PERMANONA | |
| --- | --- | --- | --- | --- |
| Comparison | R statistic | *p*-value | R^2^ | *p*-value |
| KF vs CK | 1 | 0.028 | 0.439 | 0.032 |
| MRF vs CK | 1 | 0.03 | 0.538 | 0.001 |
| MRF vs KF | 0.906 | 0.032 | 0.312 | 0.029 |
| CMF vs CK | 1 | 0.031 | 0.472 | 0.001 |
| CMF vs KF | 0.161 | 0.133 | 0.157 | 0.074 |
| CMF vs MRF | 0.932 | 0.025 | 0.325 | 0.001 |
| F vs CK | 1 | 0.022 | 0.455 | 0.026 |
| F vs KF | 0.594 | 0.029 | 0.246 | 0.026 |
| F vs MRF | 0.854 | 0.029 | 0.304 | 0.03 |
| F vs CMF | 0.662 | 0.034 | 0.269 | 0.022 |

R statistics represent difference of mean ranks between the two groups. Values closer to 1.0 indicate greater dissimilarity between the two groups compared.

R^2^ values present the proportion of variation constrained by treatments.
